# A Rare presentation of neurobrucellosis in a child with Recurrent transient ischemic attacks and pseudotumor cerebri (A case report and review of literature)

**Published:** 2014

**Affiliations:** 1 Department of Pediatric Neurology, Ghaem Medical Center, Mashhad University of Medical Sciences, Mashhad, Iran

**Keywords:** Neurobrucellosis, Transient ischemic attack, Pediatric, Pseudotumor cerebri

## Abstract

Brucellosis is a multi-system infectious disease that presents with various manifestations and complications. Neurobrucellosis is an uncommon but serious presentation of brucellosis that can be seen in all stages of the disease. High index of suspicion, especially in endemic areas is essential to prevent morbidity from this disease.

The case was an 11- year -old female patient who was admitted with a severe headache that was worsening over a period of 2 months. The day after each attack, she experienced transient right hemiparesia that was lasting less than one hour (TIA) as well as blurred vision and bilateral papilledema. Laboratory findings revealed serum agglutination Wright test positive at 1/320 and 2ME test positive at 1/160. A lumbar puncture showed a clear CSF with increased opening pressure (32 cmH2O), CSF examination was within normal range (pseudotumor cerebri).To our knowledge, there has been no report for recurrent TIA in pediatric neurobrucellosis in the base of pseudotumor cerebri.

In endemic areas like Iran, unexplained neurological signs or symptoms should be evaluated for brucellosis.

## Introduction

Human brucellosis is a zoonotic granulomatous disease that is endemic in many

parts of the world, including Middle Eastern and Mediterranean countries.

Humans are affected through the consumption of unpasteurized milk, its products, and aerosolization (1,2). 

Diverse and non-specific clinical manifestations make the diagnosis difficult. The most frequent symptoms are fever, myalgia, malaise, arthralgia, weight loss, and night sweats (3).

Neurologic findings of brucellosis occur in less than 5% in adolescents (2,4,5); but its prevalence in pediatric is less than 0.8-1% of cases (6,7). 

Neurobrucellosis can present with acute or chronic encephalitis, meningitis, radiculoneuritis, myelitis, brain abscess, epidural abscess, subarachnoid hemorrhage, granuloma, cranial nerve involvement, and neuropsychiatric symptoms (4,5,8,9).

The most frequent presentations of neurobrucellosis are meningoencephalitis and meningitis, which can occur early in the course of the disease or as a late manifestation (10,11,12).

The other rare manifestations of neurobrucellosis include diabetes insipidus (13,14), cerebral venous thrombosis (15), Guillain-Barre syndrome (16), and subdural hemorrhage (17).

Its mortality rate is 0-5.5% but permanent neurologic impairments, especially deafness are common (11).

Analysis of CSF reveals an elevated protein concentration, moderate leukocytosis, and hypoglycorrhachia (10,11).

CSF and blood cultures can be negative. Thus, the diagnosis is made by detecting brucella antibodies in CSF. This is diagnostic (18).

We report an 11- year- old female patient with recurrent right hemiparesia, severe headaches, transient visual impairment, and bilateral papilledema on fundoscopic examination that has rarely been reported as a manifestation of neurobrucellosis in pediatrics.

## Case report

An 11- year- old female patient was hospitalized with a severe headache complaint that was worsening over a period of 2 months. Frontal headaches were pulsatile and were accompanied with photophobia and phonophobia. Headaches were followed by vomiting and the day after, transient right hemiparesia that lasted less than one hour. These migraine like attacks, were happening twice a week in the previous two month period. Her headaches had been treated as migraines and showed no improvement. Despite the headaches, she complained of transient blurred vision, malaise, weakness, bone pain, and weight loss during the same period. 

Two weeks before admission to the pediatric neurological department, she developed a high nocturnal fever, decreased level of consciousness, aphasia, and right hemiparesia. She was hospitalized afterward and while waiting for laboratory results, treatment started with ceftriaxone, vancomycin, and acyclovir.

In spite of responsiveness to the treatment after 3 days and increasing level of consciousness, she was referred to our department for further evaluation. On the following day, she developed recurrent right colonic focal seizures and yelling attacks that were controlled by phenytoin. 

She had a history of consumption of raw milk products. It was also disclosed that her grandfather had been treated for brucellosis 3 years ago. 

During a general physical examination, she was confused but her vital signs were stable. In neurological examinations, there was no neck stiffness, no Kernig’s or Brudzinski’s signs, and cranial nerves were intact.

A decreased force of her right limbs was observed. An ophthalmological examination was unremarkable except for severe bilateral papilledema that was more prominent in the supranasal regions.

Differential diagnoses that were considered for this patient were collagen vascular diseases, malignancies, mitochondrial disorders, and chronic inflammations. 

A rheumatologic profile especially for exclusion of systemic lupus erythematosus was requested and it was unremarkable. 

Hematologic, liver, and renal tests were within normal limits. Bone marrow aspiration and a biopsy were performed to rule out malignancy and there were no pathologic results. A tuberculin test was unremarkable. Laboratory findings revealed serum agglutination Wright test positive at 1/320 and 2ME test positive at 1/160.

A lumbar puncture was performed one day after the initiation of antibiotic treatment and after a brain MRI was performed. It showed a clear CSF with increased opening pressure (32 cmH2O). In a CSF examination, there were no leukocyte and the protein levels were within the normal range. However, increased levels of lactate (30 mg/dl, normal range up to 20) and hypoglycorrhachia (20 mg/dl, simultaneous blood glucose was 90 mg/ml) were noted. Cytology was negative for malignancy. CSF serologic tests for brucellosis were positive at 1/20. CSF and blood cultures and gram staining were negative. A brain MRI with diffusion weighted sequences revealed no abnormal results.

Acetazolamide was administered to decrease the patient’s intracranial pressure.

With the view of neurobrucellosis, the patient was placed on gentamicin, rifampin, and doxycycline. 

Twenty-eight days after the initiation of the treatment, gentamicin was changed to co-trimoxazole. This combination was continued for up to 3 months. The patient responded well to therapy and hemiparesia, aphasia, and visual impairment were resolved without any squeals. Serologic tests were repeated 2 weeks later and revealed decreasing levels of serum agglutination titers to 1/40. One month later, papillary edema was no longer detected.

On a 6 month follow up, there were no reports of relapse. 

## Discussion

The main clinical presentations of neurobrucellosis in pediatrics are demonstrated as meningoencephalitis or acute meningitis. Our patient presented with migraine like attacks, recurrent hemiparesia, blurred vision, and pseudotumor cerebri like manifestations, and, ultimately, was hospitalized for acute meningoencephalitis. 

In our patient, in addition to the clinical manifestations, the diagnosis was confirmed by positive serologic titers for brucella in serum and CSF.

Salih researched the inflammatory risk factors of pediatric stroke and only 2out of 18 children were observed to have neurobrucellosis that presented with left hemiparesia and left facial nerve palsy (19). Sayyahfa et al. have also reported a nine- year- old female patient with sudden onset of hemiparesis and aphasia that recovered after one month (20).

It has been proposed that TIA in neurobrucellosis may be due to cerebral vasospasm, infectious vasculitis, or thromboembolism (9,21,22,23).

Our patient had recurrent migraine like headaches followed by hemiparesia that occurred a day after and lasted less than 1 hour. These transient ischemic attacks were repeated more than 10 times in the previous 2 month period and they were misdiagnosed as migraine headaches. According to the normal results of MRI that was performed between attacks, probably the TIA in our patient was due to thromboembolism.

In the literature, the association of optic neuropathy and pediatric neurobrucellosis is a rare complication (24,12).

Ischemic vasculitis and inflammatory changes in the optic nerve may explain the presence of secondary optic neuropathy in neurobrucellosis (24). 

Rare visual manifestations of neurobrucellosis in children that were published in the literature were bilateral and unilateral blindness (24,26), optic neuritis (27), gradual loss of vision, and diplopia (28).

Papilledema has been reported in neurobrucellosis. 

Both optic neuritis and pseudotumor cerebri have been involved in its pathophysiology (9, 29).

Optic neuritis characterized by papilledema, painful eye movements, quick loss of visual acuity, and relative afferent pupillary defect (30). Pseudotumor cerebri presents as papilledema, increased CSF pressure, but generally preserved vision and pupillary reflexes. 

Impermanent blurred vision and diplopia (secondary to the abducent nerve dysfunction) may occur (31).

A study in 2007, described a 14- year -old female patient with pseudotumor cerebri like presentations. Strabismus in associated with bilateral abducent nerve involvement and bilateral papilledema were noted. On a follow up visit six months later, the patient had no symptoms and signs; and papillary edema was relieved (32).

Our patient had transient blurred vision on some days, which may be secondary to pseudotumor cerebri. Visual impairment and papilledema were resolved one month after the initiation of the treatment.

To our knowledge, recurrent TIA, in the base of pseudotumor cerebri, has not been reported in pediatric neurobrucellosis. 

A CSF examination of our patient revealed an increased level of lactate and hypoglycorrhachia without any pleocytosis or elevated protein. 

Habib et al. has reported an 8- year- old male patient with brucella meningitis, Jarisch-Herxheimer-like reaction, increased CSF lactate, and pleocytosis (7). 

Because of parental disagreement, an LP was not repeated. Furthermore, there is a report that demonstrated persistent hypoglycorrhachia in spite of clinical improvement in neurobrucellosis, which may not be useful to evaluate response to therapy (33).

In our patient, increasing level of consciousness after 3 days of treatment with ceftriaxone and vancomycin was notable. A high concentration of ceftriaxone in CSF may indicate its significant potency against the pathogen. Some studies have shown the efficacy of adding ceftriaxone to the antibiotic treatment regimen of neurobrucellosis. However, our pediatric infectious consultant preferred changing ceftriaxone to rifampin, doxycycline, and gentamicin. 

Erdem et al. showed ceftriaxone based protocols are more successful than oral treatment regimens for neurobrucellosis (34). Gul et al. reported successful management of neurobrucellosis with ceftriaxone in seven patients (3).

Even though, there is not enough data regarding the routine use of ceftriaxone in the management of CNS brucellosis, it seems to be practical for pediatric neurobrucellosis. 

**In conclusion, **diagnosis is difficult because of the variety and non-specificity of the clinical presentations of neurobrucellosis. Clinical awareness and consideration of neurobrucellosis in patients with unexplained neurological impairment, especially in endemic areas, is important to reduce morbidity and mortality from this disease.
